# Adsorption Behavior of Selective Recognition Functionalized Biochar to Cd(II) in Wastewater

**DOI:** 10.3390/ma11020299

**Published:** 2018-02-14

**Authors:** Shiqiu Zhang, Xue Yang, Le Liu, Meiting Ju, Kui Zheng

**Affiliations:** 1 College of Environmental Science and Engineering, Nankai University, Tianjin 300350, China; 1120170189@mail.nankai.edu.cn (S.Z.); 2120170638@mail.nankai.edu.cn (X.Y.); 2 Analytical and Testing Center, Southwest University of Science and Technology, Mianyang 621010, China; zhengkui@swust.edu.cn

**Keywords:** biochar, targeted adsorption, Cd(II), adsorption

## Abstract

Biochar is an excellent absorbent for most heavy metal ions and organic pollutants with high specific surface area, strong aperture structure, high stability, higher cation exchange capacity and rich surface functional groups. To improve the selective adsorption capacity of biochar to designated heavy metal ions, biochar prepared by agricultural waste is modified via Ionic-Imprinted Technique. Fourier transform infrared (FT-IR) spectra analysis and X-ray photoelectron spectroscopy (XPS) analysis of imprinted biochar (IB) indicate that 3-Mercaptopropyltrimethoxysilane is grafted on biochar surface through Si–O–Si bonds. The results of adsorption experiments indicate that the suitable pH range is about 3.0–8.0, the dosage is 2.0 g·L^−1^, and the adsorption equilibrium is reached within 960 min. In addition, the data match pseudo-second-order kinetic model and Langmuir model well. The computation results of adsorption thermodynamics and stoichiometric displacement theory of adsorption (SDT-A) prove that the adsorption process is spontaneous and endothermic. Finally, IB possesses a higher selectivity adsorption to Cd(II) and a better reuse capacity. The functionalized biochar could solidify designated ions stably.

## 1. Introduction

It is well known that farmland, an important section of ecological environment, is closely related to the problems of resource, grain and environment [1,2,3]. However, with the development of industrialization and urbanization of China, the total area of heavy metal (such as Cd, As, Pb, Hg, Zn, etc.) contaminated soil increases rapidly and is more than 2 × 10^5^ km^2^, occupying about 1/5 of the total agricultural area [4]. Meanwhile, the research of the Ministry of Agriculture of the People’s Republic of China indicated that the main reasons for the contaminated soil were wastewater irrigation and sludge application [5], which could cause for crop failure up to 1 × 10^7^ t and lead to total economic loss surpassing $2.5 billion. Among all the heavy metal ions, cadmium is the most higher poisonous substance [6], jeopardizing the health of humans and animals through the food chain system of water–soil–plant–animal–human [7]. The migration capacity of cadmium was higher than the other chemical elements from water to humans and animals [8,9]. Once cadmium enters the human body through the respiratory tract or digestive tract, it could destroy the tissues and organs [10]. Consequently, the development of reliable methods for the removal of cadmium from environment and biological samples was particularly significant.

For scavenging contaminants, the methods of biological treatment, adsorption, precipitation, membranes separation and ion exchange had been carried out [11,12]. Among these methods, the effect of biological treatment was slow and precipitation method could cause the new contaminants. For removing cadmium from dilute solution, the adsorption technique possessed of a better applicability than traditional extraction process [13]. In recent years, with the discovery of black earth in Amazon Basin and the development of correlational research [14], biochar has gained much publicity as a new type of environmental functional material. Many studies indicated that biochar has huge application potential in the matter of the reduction of greenhouse gas emissions, agromelioration, and contaminated soil remediation [15,16,17]. Among these advantages, biochar has a strong capacity to absorb heavy metal ions [1,17], such as Cd(II), Pb(II), Cu(II) etc., and reduce the effectiveness and migration of heavy metal ions in the wastewater. Furthermore, the raw material sources of biochar are extensive, e.g., agricultural wastes (wood, straw, or shell), municipal solid wastes (refuse and sludge), and other organic materials [18]. In addition, biomass solid waste is a big problem. The biomass waste brings not only water, atmospheric and soil pollution, but also the safety problem of humans and animals via the food chain. Hence, the comprehensive utilization of biomass is also a crucial challenge. Moreover, biochar could adsorb contaminants simultaneously which may reduce the contents of beneficial components in the water. Ionic-Imprinted Technique is similar to Molecular-Imprinted Technique [19,20], and can recognize metal ions after imprinting. The effectiveness of the materials in binding metal ions has been attributed to the complexation between the ligand and the metal ions. The specificity of a particular ligand toward target metal ions is the result of a conventional acdi–base interaction between the ligand and the metal ions. Some of these sulfydryl-functionalized sorbents could exhibit specific interactions with soft Lewis acids (e.g., Hg(II), Cd(II), Cu(II), or Ag(I)), and the selectivity of these materials is usually remarkable because many metals have the ability to bind with thiol ligands considering the stereochemical interactions between the ligand and metal ions [21,22]. An efficient adsorption material should consist of a stable and insoluble porous matrix with suitable active groups—typically organic groups interacting with heavy metal ions. In addition, the imprinted polymers could not only possess of better selective adsorption capacity, but also be reused at least 100 times without loss of affinity towards the template ions under acidic and basic conditions, and an elevated temperature [23]. Quartz dispersed on the biochar surface were often accompanied by hydroxyl under hydrolysis. The quartz offered the action sites to the modifier [24,25]. Thus, it is a good idea to make use of the characters to modify biochar.

The objective of this work is to explore the selective recognition performance of a biochar prepared by green waste using 3-Mercaptopropyltrimethoxysilane as the surface conditioning agent and Epoxy-chloropropane as the cross-linking agent via Ionic-imprinted Technique. To investigate the adsorption capacity and selective recognition performance of the selective recognition functionalized biochar (IB), the initial pH of Cd(II) solution, sorbent dosage, adsorption kinetics and adsorption thermodynamics are studied, and analyzed by X-ray diffraction (XRD) patterns, Fourier transform infrared (FT-IR), Zeta potential analysis and X-ray photoelectron spectroscopy (XPS).

## 2. Experiment

### 2.1. Materials and Reagents

Biochar (80 mesh, green waste under slow pyrolysis at 600 °C for a retention time of 10 h) was used in this study as the substrate material to prepare the ion-imprinted functionalized sorbent. All chemicals were analytical grade, and the water used in all experiments was deionized water (18.25 MΩ·cm). 3-Mercaptopropyltrimethoxysilane (MPS, Heowns Chemical Factory, Tianjin, China), Epoxy-chloropropane (ECH, Kemiou Chemical Reagent Co. Ltd., Tianjin, China), and CdCl_2_·2/(5H_2_O) (Aladdin Chemical Reagent Co. Ltd., Shanghai, China) were used in this study.

### 2.2. Biochar Modification

Exactly 4.0 g biochar (100 mesh) mixed with 200 mL 6.0 mol·L^−1^ hydrochloric acid were stirred for 8 h, then the solid product was recovered via filtration, and washed by deionized water to pH = 6.0, and dried under vacuum at 70 °C for 8 h. The sample was denoted as activated biochar (AB).

**Figure 1:**
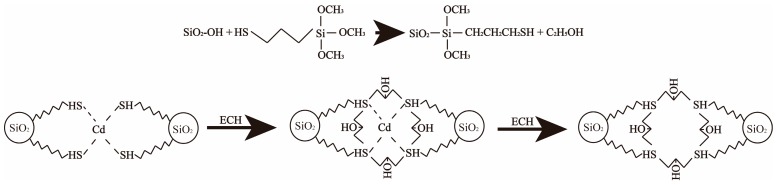
Preparation procedure of IB.

Exactly 1.92 g CdCl_2_·2/(5H_2_O) were dissolved in 80 mL alcohol (95%) with stirring and heating at 50 °C, and then 4 mL MPS was injected in and reacted for 2 h. Next, 10.0 g activated biochar was added in the mixed solution, and the mixture was reacted for 20 h at 80 °C under stirring, recovered by filtration and washed 3 times with ethanol. Then, the sample was stirred for 2 h in 100 mL 6 mol·L^−1^ hydrochloric acid, and recovered via filtration, washed by 0.10 mol·L^−1^ NaHCO_3_ and deionized water up to the eluent pH = 6.0–7.0, dried under vacuum at 80 °C for 12 h, and denoted as imprinted biochar (IB). For comparison, the non-imprinted functionalized biochar was also prepared using an identical procedure, but without the addition of CdCl_2_·2/(5H_2_O), and denoted as non-imprinted biochar (NIB). The preparation procedure of IB is shown in Figure 1.

### 2.3. Adsorption Experiments

The methodology for adsorption experiments is as follows. All adsorption experiments were executed using an air thermostatic shaker (HNYC-2102C, Honour Instrument, Tianjin, China), including the factors of initial pH of Cd(II) solution, sorbent dosage, contact time, initial Cd(II) solution concentration and adsorption temperature. The effect of initial pH of Cd(II) solution was carried out by dispersion of 0.1000 ± 0.0002 g IB and 50.00 mL 0.10 mmol·L^−1^ Cd(II) solution in 100 mL conical flask. The pH of Cd(II) solution was adjusted to the range of about 1.0–14.0 by 0.1 mol·L^−1^ HCl or 0.1 mol·L^−1^ NaOH solutions, and then the mixtures were agitated at 298.15 K for 1440 min. Afterwards, to investigate the effect of sorbent dosage, the IB dosage was changed from 0.5 to 5.0 g·L^−1^ with 50.00 mL 0.10 mmol·L^−1^ Cd(II) solution in 100 mL conical flask which pH about 5.0–6.0. The adsorption kinetics was determined by analyzing adsorption capacity at different time intervals (5–1440 min) with the same sorbent dosage (2.0 g·L^−1^) and initial Cd(II) concentration (0.1 mmol·L^−1^, 50.00 mL) in 100 mL conical flask with pH about 5.0–6.0. For adsorption isotherms, different concentrations of Cd(II) solution (0.02–0.10 mmol·L^−1^, 0.02 mmol·L^−1^ interval, 50.00 mL) were agitated until equilibrium was achieved with the same sorbent dosage (2.0 g·L^−1^) in 100 mL conical flask with pH about 5.0–6.0. The temperature factor was investigated by determining the adsorption capacity at 298.15 K, 303.15 K, and 308.15 K. In all adsorption experiments, the mixtures were separated by 0.45 μm filter membrane and the Cd(II) concentrations were measured by inductively coupled plasma-atomic emission spectrometry (ICP-AES) (iCAP6500, Thermo fiShher scientific, Franklin, KY, USA).

The selective recognition adsorption experiments of Cu(II), Co(II), Pb(II) and Zn(II) ions with respect to Cd(II) were conducted using IB Biochar-based (BS) and NIB. Next, 0.1000 ± 0.0002 g sorbent were added in 50.00 mL metal ions mixed solution containing 0.10 mmol·L^−1^ Cd(II)/Cu(II), Cd(II)/Zn(II), Cd(II)/Co(II) and Cd(II)/Pb(II) at pH 5.0–6.0 in 100 mL conical flask. After adsorption equilibrium, the concentration of each ion in the remaining solution was measured by ICP-AES. Cd(II) was desorbed by the treatment of with hydrochloric acid. In this section, 0.1000 ± 0.0002 g employed IB with 50.00 mL 6.0 mol·L^−1^ hydrochloric acid in 100 mL conical flask was agitated for different durations (0.5, 1, 2, 4, 8, 12, 16, and 24 h) by a magnetic stirrer. The final Cd(II) concentration in the aqueous phase was measured by ICP-AES. The ratio of desorption was calculated from the amount of Cd(II) adsorbed on IB and the final Cd(II) concentration in the desorption medium. Finally, all experimental results are the averages of thrice repeated experiments.

### 2.4. Characterizations

XRD measurements were performed using an X-ray Diffractometer (X’Pert PRO, PANalytical, Almelo, Netherlands) with a Cu-Kα (λ = 0.15418 nm) radiation source. A continuous scan mode was used to collect the 2θ scan XRD data from 5° to 70° at the scanning speed of 5°/min; the voltage and current of the source were 40 kV and 40 mA, respectively. The XPS analyses were performed using a Kratos AXIS Ultra XPS system (Shimadzu, Kyoto, Japan) equipped with a monochromatic Al X-ray source at 150 W. Each analysis started with a survey scan from 0 to 1350 eV with a dwell time of 8 s, pass energy of 150 eV at steps of 1 eV with 1 sweep. For the high-resolution analysis, the number of sweeps was increased, the pass energy was lowered to 30 eV at steps of 50 meV, and the dwell time was changed to 0.5 s. FT-IR spectra were collected in the range of 4000–400 cm^−1^ by Spectrum One (Version BM) FT-IR (PerkinElmer, Waltham, MA, USA) spectrometer with 32 scans resolution of 2 cm^−1^. Approximately 10% (mass fraction) of the solid sample was mixed with spectroscopic grade KBr. The Zeta-potentials of the fresh biochar and ageing biochar were measured using a Zetasizer Nano Zs90 (Malvern Instruments, Worcestershire, UK) at room temperature (25 °C) [26]. They were monitored continuously in terms of the conductivity and pH of the suspension during the measurement. The biochar samples were ground to a size of 2 µm using an agate mill. The suspension was prepared by adding 30 mg of biochars to 50 mL of deionized water. The prepared suspension was conditioned by magnetic stirring for 5 min, during which the pH of the suspension was measured. After settling for 10 min, the supernatant of the dilute fine particle suspension was obtained for zeta-potential measurements. Three measurements of zeta potentials were obtained, and their averages were taken as the results.

### 2.5. Data Analysis

#### 2.5.1. Adsorption Kinetics Analysis

To investigate the mechanism of Cd(II) adsorption on IB, two kinetic models [17,20,27], pseudo-first-order kinetic model and pseudo-second-order kinetic model, were tested to find the best fitted model for the experimental data.

The pseudo-first-order kinetic equation:

$$\log\left(q_{e}-q_{t}\right)=\log q_{e}-\frac{k_{1}t}{2.303}$$

where *k*_1_ is constant rate (min^−1^), and *q_e_* and *q_t_* are Cd(II) adsorption amounts (mg·g^−1^) at equilibrium *t* and time *t* (min), respectively.

The pseudo-second-order kinetic equation:

$$\frac{t}{q_{t}}=\frac{1}{k_{2}q_{e}^{2}}+\frac{t}{q_{e}}$$

where *k*_2_ is constant rate (min^−1^), and *q_e_* and *q_t_* are the Cd(II) adsorption amount (mg·g^−1^) at equilibrium *t* and time *t* (min), respectively.

#### 2.5.2. Adsorption Thermodynamics Analysis

Langmuir and Freundlich models were used to describe the adsorption process [17,20,27].

Langmuir adsorption isotherms equation:

$$\frac{C_{e}}{q_{e}}=\frac{1}{bq_{m}^{}}+\frac{C_{e}}{q_{m}}$$

where *C_e_* is the equilibrium concentration (mg·L^−1^), *q_e_* is the equilibrium capacity of Cd(II) on the IB, *q_m_* is the monolayer adsorption capacity of the sorbent (mg·L^−1^), and *b* is the Langmuir adsorption constant (L·mg^−1^).

Freundlich adsorption isotherms equation:

$$\log q_{e}=\log K_{f}+\frac{1}{n}\log C_{e}$$

where *K_f_* and *n* are the Freundlich adsorption constant which indicate the adsorption capacity and intensity, respectively; and *q_e_* is the equilibrium capacity of IB to Cd(II).

The data of adsorption isotherms were used to estimate the thermodynamic parameters, Gibbs free energy change (Δ*G*^0^), Enthalpy change (Δ*H*^0^), and Entropy change (Δ*S*^0^), calculated using the following equations:

$$\Delta G^{0}=-RT\times \ln K_{d}$$

$$\ln K_{d}=\frac{\Delta S^{0}}{R}-\frac{\Delta H^{0}}{RT}$$

$$K_{d}=\frac{q_{e}}{C_{e}}$$

where *K_d_* is the distribution coefficient, *T* is the temperature (K), and *R* is the gas constant (8.3145 J·mol^−1^·K^−1^).

#### 2.5.3. Stoichiometric Displacement Theory of Adsorption Analysis (SDT-A)

The stoichiometric displacement theory of adsorption equation is as follows.

$$\ln K_{d}=\beta -\frac{q}{Z}\log C_{e}$$

where *K_d_* (*K_d_* = *q_e_*/*C_e_*) is the partition coefficient of solvent in liquid solid phase, *C_e_* is equilibrium adsorption concentration (mg·L^−1^), *β* is a constant that measures the affinity of the solute to the sorbent, *Z* represents the total moles of the solvent released or adsorbed for 1 mol solute together with its corresponding contact area on the adsorbent surface during the adsorption or desorption process, is *q* is the reduced molecule number of the solvent.

When the temperature is invariant, *β* and *q/Z* are constant, while *β* and *q*/*Z* are linear to 1/*T*.

The definition of *β* is

$$\beta =\frac{k_{1}}{T}+b_{1}$$

The definition of *q*/*Z* is

$$\frac{q}{Z}=\frac{k_{2}}{T}+b_{2}$$

where *β* and *q*/*Z* are obtained by the slope and intercept of the straight line plotting lg*K_d_* versus lg*C_e_*.

Δ*G_T_*, Δ*H_T_*, and Δ*S_T_* are calculated as follows.

$$\Delta G_{T}=-2.303Rk_{2}\log C_{e}-2.303RTb_{2}\log C_{e}+\Delta G_{A},\Delta G_{A}=-2.303Rk_{1}-2.303RTb_{1}\;\Delta H_{T}=2.303Rk_{2}\log C_{e}\Delta H_{A},\Delta H_{A}=-2.303Rk_{1}\;\Delta S_{T}=-2.303Rb_{2}\log C_{e}+\Delta S_{A},\Delta S_{A}=2.303Rb_{1}$$

## 3. Results and Discussion

### 3.1. Biochar Characterization

#### 3.1.1. XRD Analysis

Figure S1 shows the X-ray diffraction (XRD) patterns of the initial biochar and activated biochar (AB), indicating that the diffraction peaks of the initial biochar matched well with the pattern of standard diffraction peaks of sylvite (*pdf* = No. 41-1476), quartz (*pdf* = No.46-1045), graphite (*pdf* = No. 41-1487), calcite (*pdf* = No. 05-0586) and brushite (*pdf* = No. 09-0077), respectively, and also that the main mineral in AB was quartz (*pdf* = No. 46-1045). The comparison indicates that interfering metallic materials on the carbon surface are removed and the silicon content is relatively enhanced.

#### 3.1.2. Zeta Potential Analysis

Figure S2 presents the zeta-potentials of the activated biochar and imprinted biochar. It indicates that the point of zero charges (PZCs) of the activated biochar and imprinted biochar are absent and may both locate at around pH < 2.0. The decrease in the zeta-potentials of imprinted biochar could be attributed to the specific functional groups onto the initial biochar surface, which is distributed to the –OH groups and –SH groups on the IB surface.

#### 3.1.3. FT-IR Analysis

The FT-IR spectra of MPS, AB and IB are shown in Figure S3. Compared with AB, the spectral features of MPS in IB are obtained [21,28]. The bands at 2923 cm^−1^ and 2853 cm^−1^ reflect –CH_2_ stretching vibration. A broad peak is noted at 1095 cm^−1^, due to the Si–O–Si, which shifted from 1048 cm^−1^ and indicates MPS interacted on the Si–O site on the surface of activated biochar. The absorption band at 2152 cm^−1^ is assigned to S–H vibrations of sulfydryl group.

#### 3.1.4. XPS Analysis

Table 1 presents the binding energies and relative contents of major elements on biochar surface. For C1s, the content on the initial biochar surface is 53.75%, and it increases after activating and imprinting, which is attributed to that the Mg, K, etc. ions in elution. For Si2p, after the activated biochar is treated by the MPS, the chemical shift of Si2p increases from 102.69 eV to 103.72 eV, indicating that MPS reacted on the surface of the activated biochar with Si–O forming Si–O–Si bends. For S2p, the results indicate that the content of S on the activated biochar surface is low and the bending energy is 164.41 eV. After imprinting, the content of S increases and the bending energy of S2p decreases to 163.01 eV; however, the bending energy shifts to 163.89 eV and the content increases to 3.03% after elution by hydrochloric acid, presenting that MPS acts on the surface of the activated biochar and the –SH group acts with Cd(II).

### 3.2. Adsorption Experiment Results

#### 3.2.1. Adsorption Kinetics

**Figure 2:**
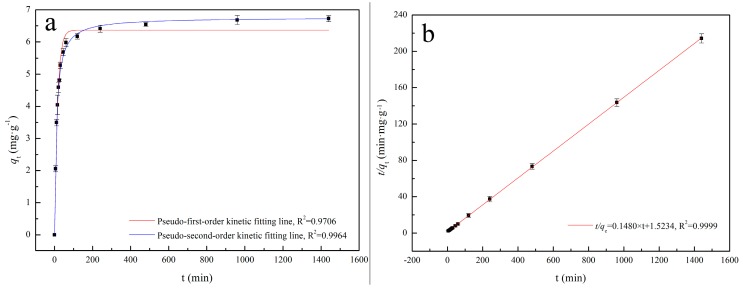
(**a**) Relation between Cd(II) adsorption amount and the contact time; and (**b**) the kinetic fitting line.

Figure 2 shows that the uptake of Cd(II) by IB is rapid during the initial 120 min and the equilibrium is reached within 960 min (*q*_960_ was similar to *q*_1440_). In Figure 2, the adsorption data match the pseudo-second-order kinetic model well (*R*^2^ = 0.9964), and the parameters of pseudo-second-order kinetics are fitted. The calculated *q_e_* (6.76 mg·g^−1^)from the pseudo-first-order kinetic model agrees very well with the experimental data. Thus, the adsorption process is a chemisorption process.

#### 3.2.2. Adsorption Isotherms

**Figure 3:**
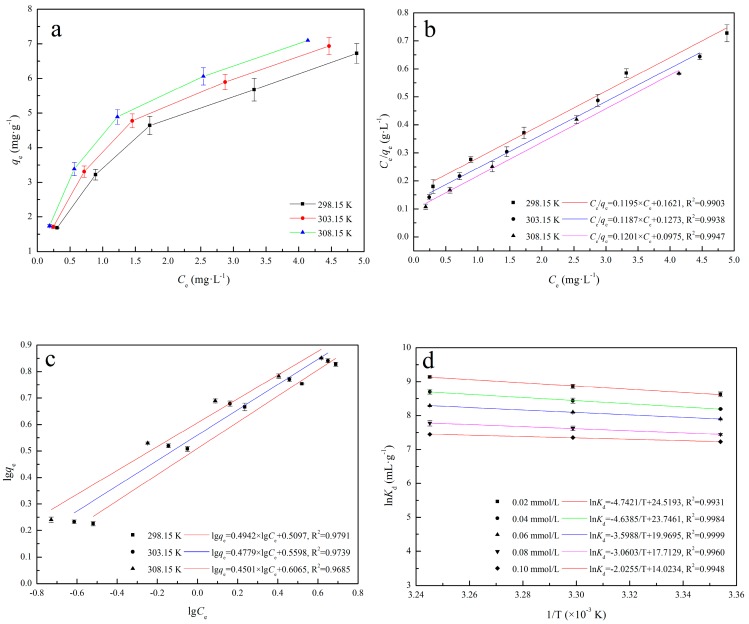
Adsorption isotherms parameters: (**a**) Adsorption isotherms of Cd(II) on IB; (**b**) The fitting line of Langmuir model; (**c**) The fitting line of Freundlich model; (**d**) Plots of ln*K_d_* – 1/T of IB.

The equilibrium adsorption isotherm is fundamental in describing the interactive behavior between solute and sorbent, and it is important for the design of adsorption system. Figure 3a shows the Cd(II) adsorption capacity on IB at different initial Cd(II) concentrations and temperature. It is evident that the initial Cd(II) concentrations affect the adsorption capacity of IB to Cd(II): the adsorption capacity of IB increase with the initial Cd(II) concentration, and adsorption favors higher temperatures.

Figure 3b,c (Langmuir and Freundlich, respectively) shows the isotherm constants and the correlation coefficients (*R*^2^) obtained by linear regression. It indicates that the adsorption process is well described by the Langmuir model ($R_{Langmuir}^{2}$ > $R_{Freundlich}^{2}$). The fact that the Langmuir isotherm fitted the experimental data very well may be due to homogenous distribution of active sites on the IB surface. Δ*S*^0^ and Δ*H*^0^ were calculated from the slope and intercept of Van’t Hoff plots of ln*K_d_* versus 1/*T* (Figure 3d). In Table 2, negative Δ*G*^0^ and positive Δ*H*^0^ indicate that the adsorption process is spontaneous and endothermic. The positive Δ*S*^0^ reflects an increase in randomness at the solid/solution interface during Cd(II) adsorption on IB.

#### 3.2.3. Stoichiometric Displacement Theory of Adsorption

However, these gas–solid adsorption equations were originally derived only for gas–solid adsorption systems, and they have not been related to the strong interactions that exist among the solute, solvent and solid sorbent in a liquid–solid adsorption process. The adsorption mechanisms of liquid–solid systems are more complex than those in gas–solid systems. In addition, the volume of the adsorption layer on solid sorbent surface cannot be estimated accurately, so the values of Δ*H*, Δ*S* and Δ*G* cannot be calculated accurately. Recently, stoichiometric displacement theory of adsorption (SDT-A) [29,30,31] has been used to explain the adsorption mechanisms of solute in liquid–solid system.

**Figure 4:**
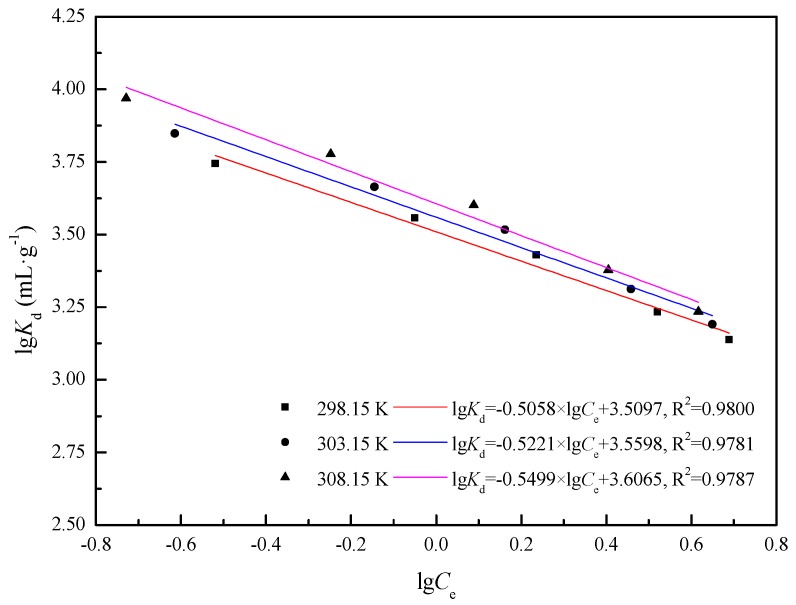
Plots of ln*K_d_* − lg*C_e_* of IB.

The data in Table 3 and Figure 4 indicate that: (1) At the same initial Cd(II) concentrations, the negative Δ*G_A_* indicates that Cd(II) adsorption on the IB was a spontaneous and heat release process, while increasing temperature benefits the adsorption. Δ*G_T_* was negative and decreases, indicating that the increasing temperature was beneficial to the Cd(II) adsorption. (2) At the same adsorption temperature, the constant Δ*G_A_* and Δ*H_A_* indicate that the adsorption process was a spontaneous and exothermic process, which was unaffected by the Cd(II) concentrations. (3) Under different conditions, the function variable of Δ*S_T_* indicated that the degree of disorder of the whole system increased. Compared to the thermodynamic parameters derived from Langmuir, the values of Δ*G* varied little, however, the values of Δ*H* and Δ*S* greatly differ.

#### 3.2.4. Effect of pH

**Figure 5:**
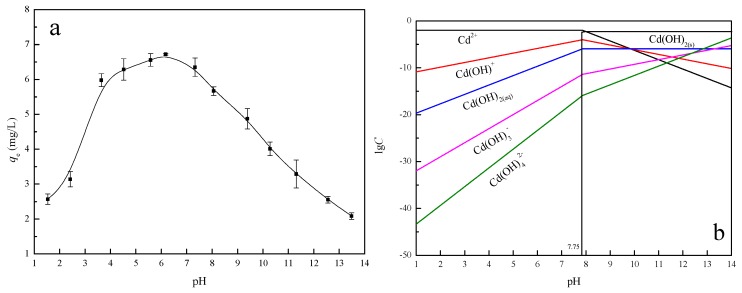
(**a**) The effect of initial pH of Cd(II) solution on the adsorption capacity of IB; and (**b**) Logarithmic diagram of Cd(II) hydrolysis components (*c* = 0.1 mmol·L^−1^).

Figure 5a presents the effect of initial pH of Cd(II) solution on the adsorption capacity of IB. It indicates that the initial pH of Cd(II) solution affects the adsorption process, especially the adsorption capacity. At the initial pH range of about 3.0–8.0, the adsorption capacity of IB to Cd(II) remains approximately stable (average about 6.26 mg·g^−1^), and there is a sharp decrease at pH > 8.0. At different pH environments, Cd(II) solution exhibited in different forms during the adsorption process, and the different forms affected the adsorption behaviors. The Cd(II) solution chemistry could be calculated to generate the concentration logarithmic diagram of each component at different pH. The relative species distribution of cadmium is calculated from the hydrolysis constants (log*β*_1_ = 4.17, log*β*_2_ = 8.33, log*β*_3_ = 9.02, log*β*_4_ = 8.62) [32]. The precipitation curve of cadmium is calculated from the precipitation constant of Cd(OH)_2(s)_ (*K_sp_* = 5.27 × 10^−15^) and the initial Cd(II) concentration (1.0 × 10^−4^ mol·L^−1^). Cd(II) is found to form precipitation at pH ≈ 7.75 (the equations are shown in the Supplementary Materials). Figure 5b shows the lg*C*-pH of the Cd(II) hydrolysis components as a function of pH when the Cd(II) concentration is 0.1 mmol·L^−1^. It indicates that the main component at pH = 3.0–8.0 is in the form of Cd(II) ion, while the main component is Cd(OH)_2(s)_ at pH > 8.0, when the reaction with IB is week. At lower pH (<3.0), the Cd(II) has to compete with hydrogen ion among the exchange sites.

#### 3.2.5. Effect of Sorbent Dosage

The effect of sorbent dosage on adsorption capacity of IB to Cd(II) is shown in Figure S4. It indicates that the sorbent dosage is an important factor to the adsorption capacity. Figure S4 shows that the removal efficiency increases from 26.05% to 78.66% when the sorbent dosage increases from 0.5 to 5.0 g·L^−1^ (Figure S5). With higher adsorbent dosage, more active sites compete for the same amount of Cd(II) ions, therefore only the higher affinity active sites will be occupied. resulting in a decrease in adsorption capacity. For a constant initial Cd(II) concentration, the increase sorbent dosage provides more functional groups and active sites, thus leading to the removal efficiency of Cd(II) increasing. When the dosage is 2.0 g·L^−1^, the adsorption amount is 6.72 mg·g^−1^ and the removal efficiency is 73.34%; however, the removal efficiency does not change with the increasing dosage. Thus, 2.0 g·L^−1^ adsorbent is selected as the optimum dose.

#### 3.2.6. Selective Adsorption

Competitive adsorption of Cd(II)/Cu(II), Cd(II)/Zn(II), Cd(II)/Co(II) and Cd(II)/Pb(II) were investigated in their double mixture systems. Relative selectivity coefficients (*η* = *k_IB_*:*k_NIB_*) for Cd(II)/Cu(II), Cd(II)/Zn(II), Cd(II)/Co(II) and Cd(II)/Pb(II) are 6.06, 5.98, 6.81 and 8.05, respectively, and the relative selectivity coefficients (*η* = *k_IB_*:*k_BS_*) are 5.37, 5.85, 6.50 and 6.10 (Table S1).

The results indicate that IB has a higher selectivity for Cd(II), even in the presence of Co(II), Pb(II), Zn(II) and Cu(II) interferences in the same medium due to the coordination geometry selectivity of IB, which could provide ligand groups arranged in a suitable way for coordination of Cd(II). Although some ions have similar size with Cd(II) and some ions have high affinity with the ligand, IB still exhibits high selectivity for extraction of Cd(II) in the presence of other metal ions. The results also indicate that the adsorption capacity and selectivity of biochar based (BS) is low.

#### 3.2.7. Desorption and Repeated Use

Desorption of Cd(II) from IB is studied in a batch experimental set-up. The best desorption time is found to be 8 h. With a single washing, desorption ratio is up to 89.19% (0.5 h: 33.67%; 1.0 h: 48.56%; 2.0 h: 65.96%; 4.0 h: 79.33%; 8.0 h: 89.19%; 12 h: 89.67%; 16 h: 90.13%; and 24 h: 90.18%).

To verify the reusability of IB, the cycle of adsorption–desorption was repeated seven times using the same sample. The results reveal that IB could be used repeatedly without significantly losing its adsorption capacities. Adsorption capacity of IB decreased only 15.64% after five adsorption–desorption cycles (Figure S6).

## 4. Conclusions

A biochar prepared by agricultural waste was modified using 3-Mercaptopropyltrimethoxysilane Epoxy-chloropropane via Ionic-imprinted Technique. IB adsorbed Cd(II) at suitable pH of about 3.0–8.0 and dosage of 2.0 g·L^−1^. During the initial 120 min, the removal of Cd(II) from IB was rapid, and the equilibrium time was about 960 min. The adsorption process matched well with pseudo-second-order kinetic model and Langmuir model. The results of adsorption isotherms and SDT-A informed that IB adsorption capacity increased with the initial Cd(II) concentration and the adsorption temperature, and the adsorption process was spontaneous and endothermic. IB had a higher selectivity for Cd(II) in the presence of Co(II), Pb(II), Zn(II) and Cu(II), which provided ligand groups arranged in a suitable way for coordination of Cd(II). Overall, 6.0 mol·L^−1^ hydrochloric acid could remove Cd(II) from IB effectively, and the desorption ratio was up to 89.19% with a single washing. Adsorption capacity of IB decreased only 15.64% after five adsorption–desorption cycles.

## Figures and Tables

**Table 1:** Binding energies and relative contents of elements on biochar surface.

Sample	Binding Energy (eV)			Surface Atomic Composition (%)		
	C1s	Si2p	S2p	C1s	Si2p	S2p
Initial biochar	284.80	102.07	164.39	53.75	9.31	0.17
Actived biochar	284.79	102.69	164.41	65.29	13.92	0.29
No-elution biochar	284.79	103.72	163.01	67.75	12.63	2.84
Imprinted biochar	284.81	103.73	163.89	69.33	13.28	3.03

**Table 2:** Thermodynamic parameters for adsorption of Cd(II) on the imprinted biochar.

*C*_0_ (mmol·L^−1^)	Δ*H*^0^ (kJ·mol^−1^)	Δ*S*^0^ (J·mol^−1^·K^−1^)	Δ*G*^0^ (kJ·mol^−1^)		
			298.15 K	303.15 K	308.15 K
0.02	39.43	203.87	−21.35	−22.37	−23.39
0.04	38.57	197.44	−20.30	−21.29	−22.27
0.06	29.92	166.04	−19.58	−20.41	−21.24
0.08	25.44	147.27	−18.46	−19.20	−19.94
0.10	16.84	116.60	−17.92	−18.51	−19.09

**Table 3:** Thermodynamic parameters of SDT-A for Cd(II) adsorption on IB.

*C*_0_ (mmol·L^−1^)	Δ*H_T_* (kJ·mol^−1^)			Δ*S_T_* (J·mol^−1^·K^−1^)			Δ*G_T_* (kJ·mol^−1^)		
	298.15	303.15	308.15	298.15	303.15	308.15	298.15	303.15	308.15
0.02	21.05	21.70	22.68	142.83	146.21	150.29	−21.53	−22.54	−23.64
0.04	17.42	18.15	18.95	126.11	129.48	133.16	−21.19	−22.22	−22.08
0.06	15.20	15.78	16.35	115.93	118.56	121.18	−19.36	−19.94	−20.76
0.08	13.00	13.48	13.89	105.78	108.00	109.90	−18.54	−19.26	−19.97
0.10	11.69	12.00	12.25	99.78	101.19	102.35	−18.06	−18.68	−19.29

Δ*H_A_* = 17.03 kJ·mol^−1^, Δ*S_A_* = 124.33 J·mol^−1^·K^−1^, Δ*G_A_* = −20.04 kJ·mol^−1^ (298.15 K), −20.66 kJ·mol^−1^ (303.15 K), −21.28 kJ·mol^−1^ (308.15 K).

## Supplementary Materials

The following are available online at http://www.mdpi.com/1996-1944/11/2/299/s1, Figure S1: XRD patterns of initial biochar and activated biochar. Figure S2: Zeta-potentials of activated biochar and imprinted biochar. Figure S3: FT-IR spectra of MPS, activated biochar, and imprinted biochar. Figure S4: Effect of sorbent dosage on adsorption capacity of SRFB to Cd(II). Figure S5: The relation between desorption ratios and desorption time. Figure S6: The relation between adsorption-desorption cycle and absorption capacity. Table S1: The selectivity parameters of Cd(II) adsorption on IB and NIB.
