# Supplementary material for: Adsorption Behavior of Selective Recognition Functionalized Biochar to Cd(II) in Wastewater
Source: Materials (Basel). 2018 Feb 14;11(2):299. doi: 10.3390/ma11020299 (PMC5848996; doi:10.3390/ma11020299)
Supplement: Supplementary file 1 [file materials-11-00299-s001.pdf]

Supplementary

Adsorption Behavior of Selective Recognition Functionalized Biochar to Cd(II) in Wastewater

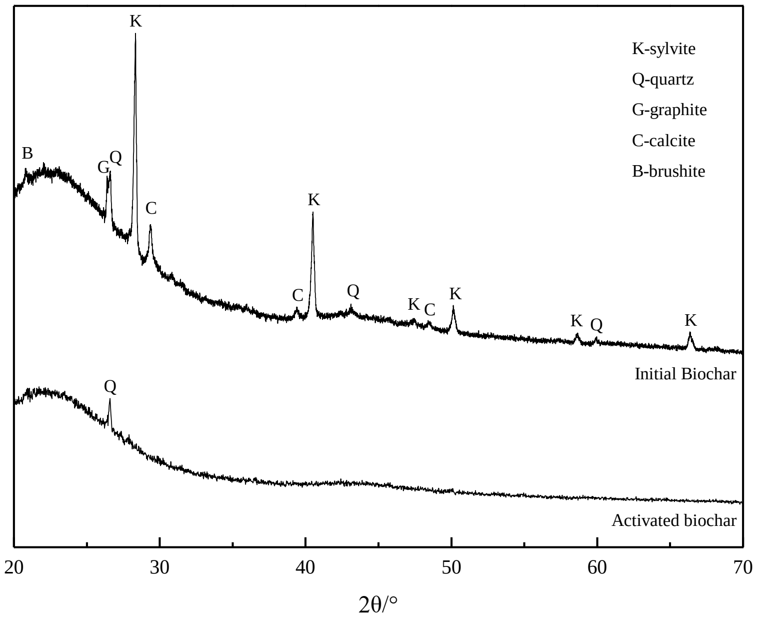

Figure S1 XRD patterns of initial biochar and activated biochar.

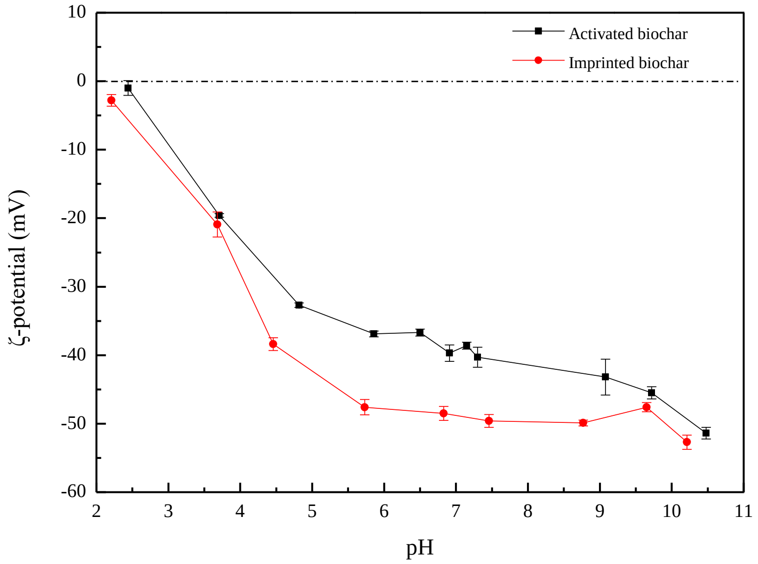

Figure S2 Zeta-potentials of activated biochar and imprinted biochar.

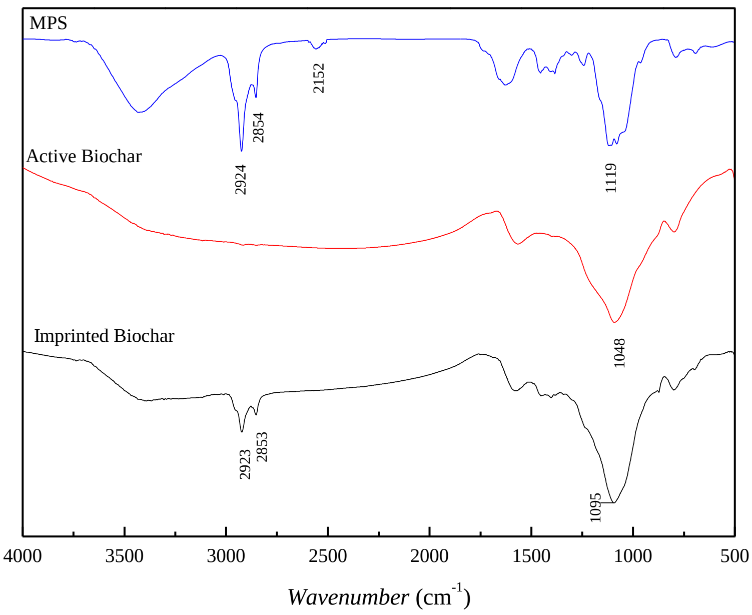

Figure S3 FT-IR spectra of MPS, activated biochar, and imprinted biochar.

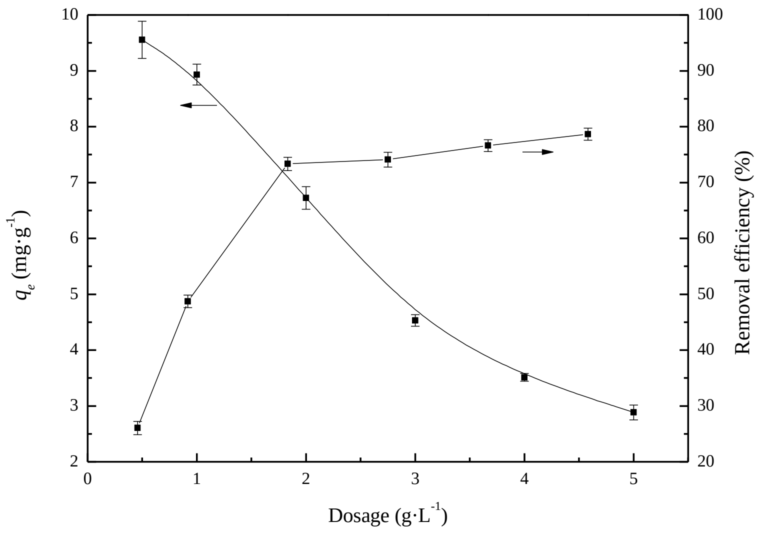

Figure S4 Effect of sorbent dosage on adsorption capacity of SRFB to Cd(II).

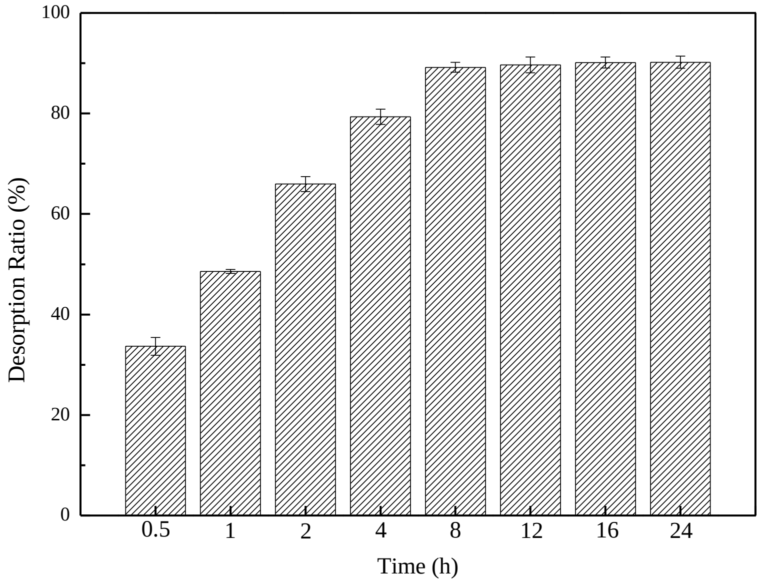

Figure S5 The relation between desorption ratios and desorption time.

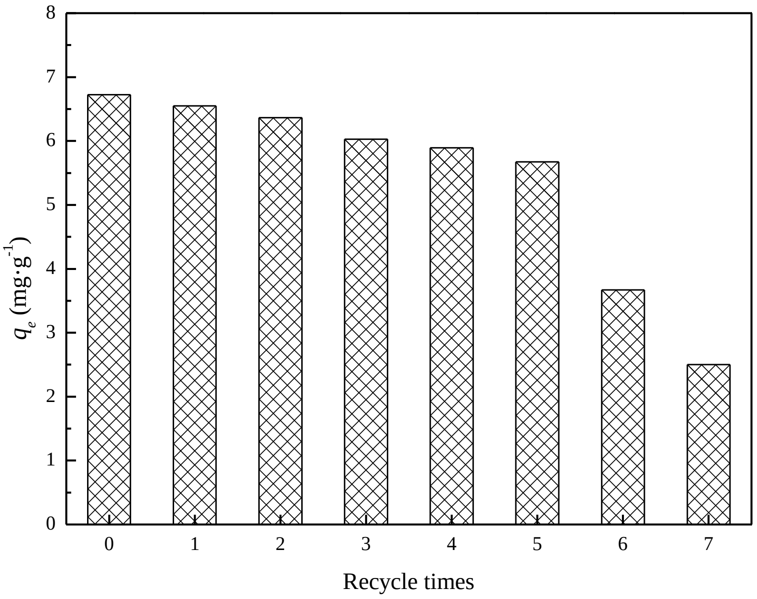

Figure S6 The relation between adsorption-desorption cycle and absorption capacity.

Table S1 The selectivity parameters of Cd(II) adsorption on IB and NIB.

**Metals Ions**	**Sorbents**	***K*d**		***k***	***η***
		**K_d (Cd)**	**K_d (Me)**		
	BS*	163.21	145.73	1.12	5.37
Cd(II)/Cu(II)	IB	1287.74	214.13	6.01	6.06
	N-IB	249.05	250.88	0.99	
Cd(II)/Zn(II)	BS	172.61	166.28	1.04	5.85

	IB	1253.71	206.20	6.08	5.99
	N-IB	189.63	186.43	1.02	
	BS	186.22	195.54	0.95	6.50
Cd(II)/Co(II)	IB	1267.82	188.95	6.71	6.81
	N-IB	209.58	212.67	0.99	
	BS	142.41	148.33	0.96	6.10
Cd(II)/Pb(II)	IB	1205.61	205.65	5.86	8.05
	N-IB	170.59	234.25	0.73	

*BS: Biochar based

Figure 5b equations

Initial Cd(II) concentration $C_t = 1\times10^{-4}$ mol·L^{-1}

$$K_{sp} = [Cd^{2+}][OH^-]^2 = 5.27\times10^{-15} \quad Cd^{2+} + 2OH^- \rightleftharpoons Cd(OH)_{2(s)}$$

So precipitation at pH≈7.75.

1. When pH is less than 7.75,

$$Cd^{2+} + OH^- \rightleftharpoons Cd(OH)^+, \quad \beta_1 = \frac{[Cd(OH)^+]}{[Cd^{2+}][OH^-]} = 10^{4.17}$$

$$Cd^{2+} + 2OH^- \rightleftharpoons Cd(OH)_{2(aq)}, \quad \beta_2 = \frac{[Cd(OH)_{2(aq)}]}{[Cd^{2+}][OH^-]^2} = 10^{8.33}$$

$$Cd^{2+} + 3OH^- \rightleftharpoons Cd(OH)_3^-, \quad \beta_3 = \frac{[Cd(OH)_3^-]}{[Cd^{2+}][OH^-]^3} = 10^{9.02}$$

$$Cd^{2+} + 4OH^- \rightleftharpoons Cd(OH)_4^{2-}, \quad \beta_4 = \frac{[Cd(OH)_4^{2-}]}{[Cd^{2+}][OH^-]^4} = 10^{8.62}$$

To:

$$lg[Cd^{2+}] = lg\,C_T - lg(1 + \beta_1[OH^-] + \beta_2[OH^-]^2 + \beta_3[OH^-]^3 + \beta_4[OH^-]^4)$$

$$= -2 - lg(1 + 10^{pH-9.87} + 10^{2pH-19.67} + 10^{3pH-32.98} + 10^{4pH-45.38})$$

$$lg[Cd(OH)^+] = lg\,\beta_1 + lg[Cd^{2+}] + lg[OH^-] = -9.87 + pH + lg[Cd^{2+}]$$

$$lg[Cd(OH)_{2(aq)}] = lg\,\beta_2 + lg[Cd^{2+}] + 2lg[OH^-] = -19.67 + 2pH + lg[Cd^{2+}]$$

$$lg[Cd(OH)_3^-] = lg\,\beta_3 + lg[Cd^{2+}] + 3lg[OH^-] = -32.98 + 3pH + lg[Cd^{2+}]$$

$$lg[Cd(OH)_4^{2-}] = lg\,\beta_4 + lg[Cd^{2+}] + 4lg[OH^-] = -45.35 + 4pH + lg[Cd^{2+}]$$

2. When pH is greater than 7.75,

$$Cd(OH)_{2(s)} \rightleftharpoons Cd^{2+} + 2OH^-, \quad K_{sp} = [Cd^{2+}][OH^-]^2 = 5.27 \times 10^{-15}$$

$$Cd(OH)_{2(s)} \rightleftharpoons Cd(OH)^+ + OH^-, \quad K_{s1} = [Cd(OH)^+][OH^-] = \beta_1 \times K_{sp}$$

$$Cd(OH)_{2(s)} \rightleftharpoons Cd(OH)_{2(aq)}, \quad K_{s2} = [Cd(OH)_{2(aq)}] = \beta_2 \times K_{sp}$$

$$Cd(OH)_{2(s)} + OH^- \rightleftharpoons Cd(OH)_3^-, \quad K_{s3} = \frac{[Cd(OH)_3^-]}{[OH^-]} = \beta_3 \times K_{sp}$$

$$Cd(OH)_{2(s)} + 2OH^- \rightleftharpoons Cd(OH)_4^{2-}, \quad K_{s4} = \frac{[Cd(OH)_4^{2-}]}{[OH^-]^2} = \beta_4 \times K_{sp}$$

To:

$$lg[Cd^{2+}] = lg\,K_{sp} - 2lg[OH^-] = -14.28 - 2lg[OH^-] = 13.72 - 2pH$$

$$lg[Cd(OH)^+] = lg\,K_{s1} - lg[OH^-] = -13.66 - lg[OH^-] = 0.34 - pH$$

$$lg[Cd(OH)_{2(aq)}] = lg\,K_{s2} = -13.36$$

$$lg[Cd(OH)_3^-] = lg\,K_{s3} + lg[OH^-] = -13.32 - lg[OH^-] = -27.32 + pH$$

$$lg[Cd(OH)_4^{2-}] = lg\,K_{s4} + 2lg[OH^-] = -13.34 + 2lg[OH^-] = -41.34 + 2pH$$

$$lg[Cd(OH)_{2(s)}] = lg(C_T - [Cd^{2+}] - [Cd(OH)^+] - [Cd(OH)_{2(aq)}] - [Cd(OH)_3^-] - [Cd(OH)_4^{2-}])$$
